# Building Low-Cost Sensing Infrastructure for Air Quality Monitoring in Urban Areas Based on Fog Computing

**DOI:** 10.3390/s22031026

**Published:** 2022-01-28

**Authors:** Ivan Popović, Ilija Radovanovic, Ivan Vajs, Dejan Drajic, Nenad Gligorić

**Affiliations:** 1 University of Belgrade, School of Electrical Engineering, Bulevar Kralja Aleksandra 73, 11120 Belgrade, Serbia; popovici@etf.bg.ac.rs (I.P.); ivan.vajs@ic.etf.bg.ac.rs (I.V.); ddrajic@etf.bg.ac.rs (D.D.); 2 Innovation Center, School of Electrical Engineering in Belgrade, Bulevar Kralja Aleksandra 73, 11120 Belgrade, Serbia; 3 DunavNET, DNET Labs, Bulevar Oslobodjenja 133, 21000 Novi Sad, Serbia; office@zentrixlab.eu; 4 Zentrix Lab, Aksentija Maksimovica 3, 26000 Pancevo, Serbia

**Keywords:** air quality, fog computing, sensor fault, microservices, management life cycle

## Abstract

Because the number of air quality measurement stations governed by a public authority is limited, many methodologies have been developed in order to integrate low-cost sensors and to improve the spatial density of air quality measurements. However, at the large-scale level, the integration of a huge number of sensors brings many challenges. The volume, velocity and processing requirements regarding the management of the sensor life cycle and the operation of system services overcome the capabilities of the centralized cloud model. In this paper, we present the methodology and the architectural framework for building large-scale sensing infrastructure for air quality monitoring applicable in urban scenarios. The proposed tiered architectural solution based on the adopted fog computing model is capable of handling the processing requirements of a large-scale application, while at the same time sustaining real-time performance. Furthermore, the proposed methodology introduces the collection of methods for the management of edge-tier node operation through different phases of the node life cycle, including the methods for node commission, provision, fault detection and recovery. The related sensor-side processing is encapsulated in the form of microservices that reside on the different tiers of system architecture. The operation of system microservices and their collaboration was verified through the presented experimental case study.

## 1. Introduction

In recent years, rapid population growth in urban environments coupled with the evolution of society is creating structural challenges for the functioning of large cities. A smart city concept tries to overcome these challenges and to satisfy the needs of citizens using modern technology. In addition to the structural problems in urban environments, there is a necessity to provide access to the citizen’s services anytime and anywhere [1]. Aside from the structural challenges, the growth of the population of cities (50% population is living in urban areas nowadays, with a prediction of 70% of the world population in 2050 [2]) consequently influences the increase in the air pollution in the city. Massive urbanization results in a strong increase in traffic (road vehicles), energy use and industrialization (80% of world CO_2_ emission and 75% of total energy consumption is related to cities [3]). A high level of air pollution is directly linked to human health, increasing associated symptoms and diseases. On the other hand, the citizens expect a better quality of life in the cities, which leads to an expectation of high air quality, among other parameters. High air quality is quite important to ensure the future development of the city, considering attracting tourists and investors that would furthermore increase its businesses growth. 

Air quality can vary on a micro-location level, due to locally predominant atmospheric flow conditions and locally specific emission sources. Air quality monitoring in the cities is mainly performed by using public monitoring stations, but due to their high cost and demanding maintenance, the number of areas covered by them is rather limited. Therefore, most of the places of interest are left out of range for air quality monitoring. In order to overcome this shortage, a network of low-cost sensors could be developed for complementary measurements which will expand spatial density and measurement resolution, thus allowing more locally traceable pollution monitoring and identification of highly polluted areas for citizens. In the paper [4], authors provided a detailed analysis of low-cost sensors’ advantages, their calibration and measurement accuracy problems. They have also presented the approach on how to obtain the tempo-spatial heterogeneity in order to identify pollution hotspots with the final aim of creating city pollution maps that provide a pollutant spatial distribution over the areas of interest [4]. In order to create highly accurate measurements and pollution maps, it is obvious that a highly dense network of monitoring devices needs to be created, keeping in mind that it should be cost-effective. The current trend in air quality monitoring is the usage of devices with low-cost sensors to create large-scale environmental monitoring deployments. On the other hand, sensors’ calibration is not done in laboratories because such a procedure would make installations prohibitively expensive. Instead, the sensors that come initially pre-calibrated from the manufacturers are then calibrated using the collocation calibration method. The obtained data might not always be precise because it is well known that the relative humidity and temperature could have a negative effect on the performance of sensors and electronic circuits, given that a sensor has its own sensitivity to these properties. Thus, appropriate correction algorithms should be developed and applied. 

Aside from the identified issues related to the sensor-side data processing—e.g., sensor calibration, correction, and accuracy—there are many challenges regarding their integration in large-scale deployment scenarios and applications, like their identification, configuration and discovery, life cycle management, etc. 

Nowadays, the implemented solutions in the domain of air quality monitoring are often based on cloud computing. However, they have expressed certain limitations regarding indeterministic latency, lack of mobility support, and location awareness. Large-scale deployment of low-cost sensors requires the integration of a substantial number of sensors, which consequently brings many challenges and open issues. In general, a centralized cloud model is not always optimal considering the data volume, communication and processing requirements of large-scale deployments. Additionally, a cloud-only solution is identified as a bottleneck in providing autonomy of operation, orchestration and management of system services.

A newly introduced fog computing concept, as an extension of the cloud-only systems, provides advanced solutions for these problems [1]. The fog computing concept extends the traditional cloud computing concept to the physical edge of the IoT network, enabling the creation of refined, scalable, time-aware and location-aware applications and services [5]. As a novel trend in computing, fog computing aims to process data near the data source, by pushing away the application services and computing resources from the centralized cloud, located at the network core, toward the logical extremes of a network. This way some of the decision-making processes could be performed at the adjacent fog nodes instead of the distant cloud core services [5]. 

In the context of Air Quality Monitoring (AQM), we adopted fog computing as a framework that is capable of addressing the majority of the concerns in building infrastructure for AQM. These concerns are not only related to information processing and data communication but also to the issues of security, availability, reliability, serviceability, openness, manageability, etc. Additionally, fog computing is helping to sustain IoT momentum and to overcome the limitations of traditional cloud-only solutions found in large end-to-end delays and bandwidth constraints. The limitations of cloud-only solutions are expected to be more noticeable in handling high velocity and large volumes of data generated by distributed IoT sensors in large-scale deployments. The introduction of fog tiers, where the data are processed near the edge of the network, is found to be beneficial to improve the quality of service of the IoT network, reducing bandwidth requirements and saving processing time and resources at the cloud end. Moreover, filtering and pre-processing of irrelevant or erroneous data results in a more efficient and computationally more reliable AQM system. Therefore, fog computing concepts provide the generalized framework to effectively solve the problems of creating sophisticated and scalable system solutions capable of meeting the requirements of the AQM as a part of a future smart city.

Our paper addresses different architectural and implementation aspects in building AQM infrastructure applicable in urban environments. Architectural aspects include the system-level architecture, deployment architecture and fog nodes software architecture. Implementation aspects include the methods for sensor side data processing and the methodology for their implementation in the form of system services. Both software architectures and the service operation are analyzed from the manageability perspective as one of the most important system concerns found in large-scale deployment. In addition, the proposed system is seen as a foundation leading toward more advanced urban planning, land use regulations, air pollution control and population exposure assessments for facing environmental challenges while promoting sustainable development in urban areas. 

Direct benefits and contributions of the presented fog-based architectural solution for building low-cost sensing infrastructure for air quality monitoring in urban areas are given, as follows:

- A fog-based implementation framework provides a scalable system solution applicable for AQM in large-scale metropolitan areas, complementary with the requirements of a smart city concept.
- The implemented metrics enable the discovery of erroneous measurements as well as sensor and other operational faults, thus improving the reliability of the information, CAQI (Common Air Quality Index) [6] estimation accuracy and the life-cycle manageability of AQM devices, including their maintenance and recovery.
- Integration of low-cost AQM with reference monitoring infrastructure improves the spatial density of CAQI measurements enabling the execution of location-aware services applicable in intelligent traffic control, control of industrial facilities, monitoring and prevention of pollution peaks, air pollutant transport and dispersion monitoring, prediction of environmental risks, etc.
- Deployment of AQM services and their real-time operation, including automatic reporting, diagnostics of sensor faults and operational irregularities as well as recovery capabilities are verified in an experimental case study, as given in Section 4.
- The introduced management model supports the successful management of edge tier operation through the different phases of the edge node’s life cycle, including the node provision, early life, functional life and node decommission phase.

This paper is outlined as follows. In Section 2, a review of the most recent studies and approaches for the utilization of low-cost sensors and their integration as a part of air quality measurement systems is provided. The details of the adopted methodology for sensor-side data processing for peak elimination, noise cancelation, sensor calibration and measurement correction, as well air quality estimation methods, are provided as a part of Section 3. The architecture descriptions, given from different viewpoints, especially from the manageability perspective, are also given in Section 3. Section 4 presents several use-case scenarios, where the operation of individual services and their collaboration was investigated during the different phases of the edge-tier node’s lifecycle. Concluding remarks and the directions for future work are given in the final Section 5.

## 2. Related Work

Building an infrastructure for air quality monitoring in urban areas introduces many issues that belong to different problem domains. Most of the research efforts were focused on the methodology for sensor-side data processing, targeting correction of measurement data, detection of sensor faults, monitoring and estimating air quality, creating pollution maps, etc. However, there is a lack of research directed to the design and implementation of the infrastructure, especially from the scalability perspective. Therefore, our paper is addressing the gap between the current research efforts and the scalable system solution applicable in large-scale deployment scenarios. To handle scalability issues, a systematic approach is required as opposed to the proprietary solution. We adopted the distributed processing model as a generalized framework capable of addressing the scalability problems, although it introduces additional complexity in the implementation of processing services. Another important factor is that, when talking about the building of the complete system, the design of infrastructure elements cannot be detached from the responsibilities of integrating, controlling and maintaining the infrastructure. This issue is particularly elevated in large-scale deployment scenarios because human interventions and manual workaround, in solving integration and maintenance requests, are not feasible. Our solution is offering a systematic framework to address all the identified issues in building air quality monitoring infrastructure. 

This section gives insight into the actual research efforts and the studies addressing the range of the problems in utilizing low-cost sensors in air quality measurements and in designing complete system solutions. 

The first part of the literature review analyzes the methodologies and approaches tied to the processing of sensor measurements and the detection of sensor faults. The operation of sensors and obtained measurements is subjected to numerous errors, and appropriate methods for error minimization and elimination should be used in the post-processing of obtained raw measurements. Recently, different statistical techniques for sensor failure and outlier detection have been analyzed [7]. To overcome measurement errors, analytical redundancy and hardware redundancy, an approach is recommended in [8]. Authors in [9] have proposed a fully automated detection of erroneous raw data and concluded that for filling the gaps in low-cost air pollution measurements, linear interpolation has the best performance. A peak (a raw value that has extraordinarily low or high values in comparison to neighboring measurements) detection and correction algorithm is proposed and verified in paper [10]. Issues regarding the existence of measurement noise, which is in most cases caused by cheap electronic components, could be resolved using a moving average filter [11]. Because most of the low-cost sensors are only factory calibrated, it is of high importance to perform an additional in situ calibration. So far, there is no exact procedure for laboratory calibration of low-cost sensors, and the laboratory calibration could also be very expensive. One of the most commonly used calibration procedures is the Environmental Protection Agency (EPA) co-location method with a linear regression algorithm, described in [12]. Additionally, measurements of low-cost sensors are very sensitive to environmental conditions such as air temperature and relative humidity; therefore, the modeling of this influence and the development of correction algorithms is also of crucial importance. Because these dependencies can be quite complex, machine learning (ML) models [13] are often used as an adequate solution. The analysis presented in [13] has shown that from different evaluated ML models, the random forest algorithm expresses the best performances, in most of the observed cases, while in some cases artificial neural networks (ANN) could improve the performance as well. In [14,15,16,17], various kinds of machine learning algorithms were proposed and evaluated for different air pollution monitoring scenarios. Due to aging, sensors gradually begin to lose their sensitivity or start to show a drift in their measurements. In order to detect these anomalies in sensor behavior, it is important to identify and follow errors’ trends. In [18], data metrics, as a set of statistical parameters including Root Mean Squared Error (RMSE), Mean Squared Error (MSE) and Mean Absolute Error (MAE), are proposed to measure, quantify and evaluate the quality of measurements.

Besides the native requirements for the processing of sensor measurements, the integration of sensors in different deployment scenarios is gathering attention. To distinguish the airport emissions from long transport emissions, the complex sensor network with 40 devices has been deployed at the London Heathrow Airport [19]. After the successful evaluation of network performances, they conclude that their approach could be used for a broad range of environmental pollution studies.

Recent studies have proposed air quality monitoring systems based on IoT platforms that provide a high number of requested parameters in real-time. The systems for air quality measurement are often cloud-based, using centralized network storage and computational power, regardless of the amount and origin of data [20,21]. The data is collected from different low-cost sensors, transmitted by a low-power and long-range communication protocol to the core of the network [20,22]. 

The solution explained in [23] presented a low-cost portable device with IoT technology that connects to the internet through a GSM module and sends all real-time measurement data to a cloud platform for further processing and storage. In [24], the air quality measurement system, composed of a distributed sensor network that has been connected to the cloud system, has been implemented. The cloud centralized computing system has been performing all data processing and analysis by applying artificial intelligence techniques in the core of the network [24]. Similarly, the cloud computing-based monitoring system using the provided data from the Raspberry Pi hardware platform with built-in Wi-Fi connectivity enables the complex analysis of different air pollutants only on a periodical basis [25]. By combining both fixed and mobile IoT sensor nodes, the study has presented different ML algorithms compared to real-world data in order to provide effective monitoring and predicting of air quality [26].

The concept of pushing computing closer to the network edge, where various sensor devices gather their data, is found as a promising approach in designing modern IoT systems and applications. For many applications, including automated machinery, home automation, self-guided vehicles and robots, it is essential for the processing to be executed locally. Toward the same line, in [27] the edge of the network has been proposed for data storage and processing, including sensor calibration, training of calibration models, etc. On the testbed deployment case in Helsinki [28], massive scale air quality monitoring has been implemented, integrating tens of thousands of sensors (CO, NO, PM, T and RH) for air quality monitoring with fine spatial resolution. They identified and evaluated calibration methods as a key challenge in the design, deployment and maintenance of devices. 

With a systematic solution for integration problems in mind, fog computing is introduced as a computing approach implemented as an intermediate platform between end devices and cloud computing data centers, where the processing is performed closer to the network edge. The fog-based approach of system design offers a scalable solution for a variety of applications with real-time and low-latency requirements, at the same time supporting location-aware processing and large-scale deployment scenarios [5,29,30,31].

As given in [32,33], the fog computing concept effectively solves the problems of big data processing, data transmission and network scalability. Furthermore, it promises increased performance, energy efficiency, reduced latency, quicker response time, and better localized accuracy for different smart city applications [34]. In order to integrate such applications and services, and to meet the real-time requirements, fog computing relies on the tiered architectural model positioned as an intermediary layer between the network core and end-devices [29,35]. 

Adopting a fog-based computing approach enables a hierarchically organized data transport with a unified integration model of locally connected end-devices, both resulting in large-scale upload capabilities in vertical communication and horizontal service integration [29].

In the context of AQM, in accordance with a high number of data generated from different types of sensors, which are usually mounted throughout a wide area or on mobile platforms, using fog computing finds its justification in the processes of AQM. A study presented in [35] proposed using fog computing to monitor the environmental parameters and response in real-time based on the decision-making process that can be done on fog nodes. As an extension of cloud computing, in [36,37], fog computing has been promoted due to its inherent property of bringing the intelligence to the proximity of the edge of the network, and the effects on the performance of the service execution. The proposed AQM system based on fog computing presented in [38] introduces a distributed fog computing layer to effectively process the air pollutants’ data sent by the sensor layer. The proposed solution is seen as a feasible solution capable of surpassing the limitations of a cloud-only solution. The proposed system utilizes a fog layer to filter the irrelevant data using pre-processing and clustering techniques in order to consume less space on the cloud layer and reduce the communication bandwidth requirements. The solution presented in [39] follows the same design pattern, where service allocation is performed on fog resources, close to the IoT sensors, allowing real-time processing and data analytics at the edge of the IoT network. The presented approach enables the design of delay-sensitive IoT applications with reduced bandwidth costs because data samples are filtered and processed near the source of the data.

Following the same decentralized processing concept, the research given in [40] presents a scalable solution for air quality monitoring, offering detection and monitoring of air pollutants. To enable large-scale deployment of prediction services, the hardware platform has been integrated with the mobile application [41]. On the other hand, targeting system mobility as a most prominent area of research interest, applicable in urban environments, the approach based on mobile nodes, has been introduced in [42]. The focus of this study is to increase the level of mobility by using the existing communication infrastructure for exchanging data with the stationary nodes. The presented solution utilizes pre-processing as a data aggregation technique, applied before the data has to be sent to the centralized nodes. In [43], the real-time air quality monitoring system has been tested in a case study applied in the metropolitan city area. The system has been designed as a low-cost and scalable solution connected to the cloud-hosted application. The developed data analytics and Artificial Intelligence (AI) have been used to identify the incident situation and potential pollution sources.

A summarized review of the proposed solution and recent studies, addressing different issues regarding the design and the development of low-cost sensing infrastructure and the methodology for air quality estimation, is given in Table 1. The selection of viewpoints, used for the classification of reviewed system solutions, was performed to reveal the critical aspects regarding the research approach, research directions and intended applications of the proposed solutions. 

The majority of reviewed system solutions given in Table 1 [20,21,22,23,24,25,26,35,36,40,43] utilize a sensor-cloud approach given as an infrastructure that allows persistent system operation using sensors as an interface between physical and cyber worlds, the cloud computing services as the cyber backbone and the internet as the communication medium. Although architecture review can be found as a part of the associated research papers [22,35,36], the research novelty in these papers is more often related to the hardware design of sensing devices and algorithms for data processing. Data processing in such a sensor-cloud approach is either given as a functionality implemented in sensing devices [20,25,40,43] or with cloud-based data services [24,26]. Rarely, this data processing is shaped as multi-stage processing [26,36], where the first step in sensor processing is performed on the sensing device or intermediary gateway device. Compared with the traditional cloud-based IoT approach, cloud-edge based architecture for AQM introduces an additional processing tier, at the same time offering enhanced system scalability [36,42]. Regardless of the design approach, neither of the solutions, reviewed in Table 1, is truly distributed, offering unlimited scalability and system performance. Additionally, from the viewpoint of building AQM infrastructure, neither of the solutions addresses system design, perceiving the manageability of the infrastructure, supporting its commission and maintenance. On the other hand, the solution, proposed in this paper, is offering a distributed processing approach where data processing methodology and its implementation are carefully tailored to fulfill the manageability requirements of the future system deployment.

## 3. Methodology and System Overview

Distributed system infrastructure and the scalable requirement impose additional challenges in the domain of design and deployment of system functionalities. This complexity burdens the implementation of sensor-side data processing services because it introduces the requirements for automated provision, commission, recovery and maintenance of the underlying infrastructure. Therefore, the methodology for sensor-side processing is seen as a part of a distributed processing framework that handles additional requirements of a system-level design. The system cross-cut concern that represents the methodology and implementation context of system design is found in its manageability perspective.

The section provides the details of the underlying methodology for sensor-side data processing and its encapsulation in the form of system services. The architecture description, given as a collection of system views, service deployment and service collaboration from the fog manageability perspective, is also given. 

Section 3.1 introduces a collection of methods used in the processing of sensor measurement data, including the methods for peak elimination, sensor calibration, correction of sensor measurements, extraction of correction model parameters, detection of sensor faults, air quality estimation, etc. Section 3.2 gives the representation of different structural aspects of system architecture, while the service deployment, operation and data flow are given in Section 3.3.

### 3.1. Methodology Overview

The causes of measurement errors can be numerous, and it is of high importance to monitor, estimate and evaluate the accuracy, reliability and quality of the obtained results. Because their influence on the output of data processing services can be significant, it is found to be important to compensate for these data points. The following section gives the details of the proposed methodology given as a collection of processing methods used as a background for building sensor-side data processing services. 

Because our previous study has proven the efficiency of the peak correction algorithm described in [10], we utilize the same approach for handling such scenarios. The peak elimination algorithm is applied to the sequence of raw sensor measurements $x_{k}\ldots x_{k+n}$, with n data points, where n is selected according to the configured time window T as $n=T/T_{s}$, where $T_{s}$ is the sensor sampling period. 

The criterion for detecting peak values is evaluated for each input sample through the expression [10]:

(1) $$\forall i,\;\;\;i\in \left\{k,\ldots ,k+n\right\},\;\frac{\left|x_{i}\right|}{\left|\bar{x}\right|}>K_{1},$$

where $\bar{x}$ is calculated as a mean value of the input data sequence while $K_{1}$ is a method parameter, whose value depends on the observed pollutant.

If the expression given by Equation (1) is fulfilled in less than $K_{2}$ consecutive data samples, the peak value in the input sequence is detected. $K_{2}$ is an additional method parameter. In the event that a peak value is detected, the replacement value is calculated as the mean value of the preceding and succeeding regular sensor reading.

As an extension of peak elimination, simple filtration of sensor data is often performed [11] because the low-cost sensor measurements can be noisy. We selected a moving average filter as a basic technique used for removing noise as a random interference from data measurements. As a simplified form of a low-pass filter, the moving average technique removes higher frequency information by simply smoothing out the original data sequence. One should have in mind that the window size is the single parameter of the moving average filter, where selecting a too-large window size leads to over-smoothing, where critical information about the time series data may be lost. On the other hand, selecting a too-small window size leads to under-smoothing, where the output data sequence can end up still being very noisy. Having this in mind, the moving average presumes a time window containing m samples of the data array $x_{k}\ldots x_{k+m}$ resulting in the filtered signal $x_{out}$ according to [44]:

(2) $$\;x_{out,k+m}=\frac{1}{m}\sum_{i=0}^{m}x_{k+i}.$$

The parameters m can be altered through the configuration of the noise elimination service.

Regardless of whether the sensors are initially calibrated or not, in order to improve measurement accuracy, sensor in situ calibration is often recommended. As a method for sensor calibration, in its actual environment, we suggest the commonly used sensor calibration based on the EPA co-location with a linear regression algorithm [12]. Co-location assumes the process where both the reference sensor and the target, nonreference sensor operate at the same time interval and at the same location under the same conditions. In our case, reference sensor measurements are obtained from the reference area node, based on the reference air quality station located in the target area, other nearby sensors attached to the same area node, and their geo-location info. Thus, our method uses the low-cost pollutant measurements $X\;\left(x_{k}\ldots x_{k+n}\in X\right)$ and the estimated reference pollutant measurements $X_{ref}\;\left(x_{ref,k}\ldots x_{ref,k+n}\in X_{ref}\right)$, both of length n, to evaluate the linear regression parameters a and b according to the equation [45]:

(3) $$a=\frac{n\sum \left(xx_{ref}\right)-\sum x\sum x_{ref}}{n\sum x^{2}-{\left(\sum x\right)}^{2}},$$

(4) $$b=\frac{\sum x_{ref}}{n}-a\frac{\sum x}{n}.$$

Parameters a and b, are later used to correct any future low-cost measurement x, obtaining a calibrated measurement $x_{out}$ [45]:

(5) $$x_{out}=ax+b.$$

The length *n* of the co-location interval, used for creating the calibration dataset (X, $X_{ref}$), is a part of the configuration of the sensor calibration service that encapsulates the proposed method. 

As given in [13], low-cost sensors’ measurements are highly influenced by environmental conditions, so the modeling of this influence and the correction of the sensor measurements are of special interest. Because linear regression is a method of modeling the linear relationship between the observed datasets, in order to compensate for the influence of meteorological conditions on the low-cost sensor measurements, which are complex dependencies, a more sophisticated ML model is required. Each selected ML model comes with its own set of parameters and hyperparameters, which can be set up as a part of the related service configuration or model training process. 

In order to compensate for the influence of meteorological factors on low-cost sensor measurements, an ML technique is used in this paper. This process will be referred to as the correction of sensor measurements, and it will represent an implementation of the random forest (RF) algorithm [46] as a simple, fast and flexible tool for addressing different regression problems. For the selected set of RF hyperparameters, found during the cross-validation process, an RF model is trained using a time series of calibrated sensor measurements $x_{k}$…$x_{k+n}$, temperature and relative humidity data as inputs and reference measurements as outputs. All mentioned time series are of the same length, i.e., each data point contains information about the four mentioned variables. The different types of input variables used in the training process are called features (calibrated sensor measurements, temperature and relative humidity), and the number of features can be higher or lower than the default value of 3, based on the availability of environmental data. The number of gathered samples corresponds to the duration of the training interval.

The RF represents an ensemble of decision trees and the training process of an RF algorithm on a dataset X with N instances, and an input feature set S (includes calibrated sensor measurements, relative humidity and temperature data by default) is as follows.

Creating N subsets of X, each with m timepoints with all available features, one subset for each of the N decision trees,

(6) $$\left(X^{i},\;X_{ref}^{i}\right)\;i\in \left\{1,\ldots ,N\right\},$$

Creating a subset of the feature set S for each decision tree,

(7) $$S_{i}\;\;i\in \left\{1,\ldots ,N\right\},$$

Training each decision tree, $h^{i}\;i\in \left\{1,\ldots ,N\right\}$ on its respective

(8) $$(X_{S_{i}\;}^{i},\;X_{ref}^{i}),$$

The prediction of the random forest for a given x is

(9) $$\frac{1}{N}\sum_{i=1}^{N}h^{i}\left(x_{S_{i}}\right).$$

To reiterate, each decision tree is trained on a subset of all data points, and on a subset of features, with the output of the entire RF being the average of all decision trees. This method is called bagging, and it involves averaging a number of individual “weak learners” (simple algorithms) that are trained on subsets of all available data to provide an accurate overall prediction. Hyperparameter tuning of the RF relies on experimental model evaluation based on the gathered direct sensor measurements and the estimated reference values.

Considering a wide range of possible sensor faults, for the detection of sensor faulty operation, we selected a correlation analysis based on the monitoring of two statistical parameters, RMSE and $R^{2}$ [47]. Correlation analysis tracks the relationship between the low-cost and reference sensor measurements. One should have in mind that reference measurements are not directly observed but rather estimated based on the measurements of other neighboring sensors and the reference air quality station located in the target area according to the equation:

(10) $$x_{ref,i}=\sum_{k}w_{k}x_{i,k},$$

where $x_{i,k}$ being the sensor readings obtained from the same area, for sensors placed in the vicinity of the location of the observed sensor, and $w_{k}$ being the weights that are assigned based on the distance from the observed sensor’s geo-location (regulated by index k).

The parameter RMSE is calculated as

(11) $$\mathrm{RMSE}=\sqrt{\frac{1}{n}\sum_{i=1}^{n}{\left(x_{i}-x_{ref,i}\right)}^{2}},$$

and the parameter r is calculated as

(12) $$r=\frac{C\left(x,x_{ref}\right)}{\sigma_{x}\sigma_{x_{ref}}},$$

where C is the covariance of $x,\;x_{ref}$ and $\sigma_{x}$, $\sigma_{x_{ref}}$ are variances of x and $x_{ref}$, respectively.

The RMSE quantifies the exact Euclidean distance between the two signals, and the $R^{2}=r^{2}$ explains the agreement of trends of the predictor variable and the response variable. The criterion for detecting sensor faulty operation is evaluated during the selected sliding time window with the number of samples n (duration $T=nT_{s}$), thus taking into the consideration a time series of data $X\;\left(x_{k}\ldots x_{k+n}\in X\right)$ and $X_{ref}\;\left(x_{ref,k}\ldots x_{ref,k+n}\in X_{ref}\right)$ with n samples each. The sensor faulty operation is detected if the following logical expression is fulfilled:

(13) $$R^{2}\left(X,X_{ref}\right)<t_{R^{2}}\;\;\vee \;\mathrm{RMSE}\left(X,X_{ref}\right)>t_{\mathrm{RMSE}}\;$$

where parameters n (and therefore T*)* are defined through the selected time window, while $t_{\mathrm{RMSE}}$ and $t_{R^{2}}$ are threshold values defining the tolerances of statistical parameters. All parameters are part of the configuration of the appropriate service that encapsulates the fault detection functionality.

Besides the sensor faults resulting from the changes in the sensor behavior, there are other operational faults that denote problems in sensor connection, data communication, power supply or sensor mechanical damage. These sensor operational faults can be observed through simple criteria where missing data in the predefined time interval or catching the sensor measurements that are out of the predefined range can trigger different types of the alarms. 

Apart from processing sensor readings and detecting faults in sensor operation, sensor-side processing implies monitoring of air quality as its primary concern during regular sensor operation. In order to present the air quality index to the general public since 2006, the CAQI has been used in Europe [6,48]. According to the adopted methodology for the quantification of air quality, the level of pollution is divided into five categories (1–5), i.e., in five different colors (green to red) visually understandable to the observers. In our system, the following pollutants are observed: CO, NO_2_, SO_2_, O_3_ and PM10 on an hourly basis. CAQI of air pollution is categorized as very low, low, medium, high and very high. For each pollutant *p*, five boundary values for five ranges of values are given as $r_{P}\left(i\right)$, and the CAQI index $I_{P}$ given as i∈{1, 2, 3, 4, 5} is estimated based on the measurement $m_{P}$ as follows:

(14) $$I_{P}=i,\;r_{P}\left(i\right)\leq m_{P}<r_{P}\left(i+1\right).$$

As the $r_{P}$ values are predefined for each pollutant, no configuration is needed for the corresponding CAQI calculation service.

### 3.2. AQM System Architecture

**Figure 1:**
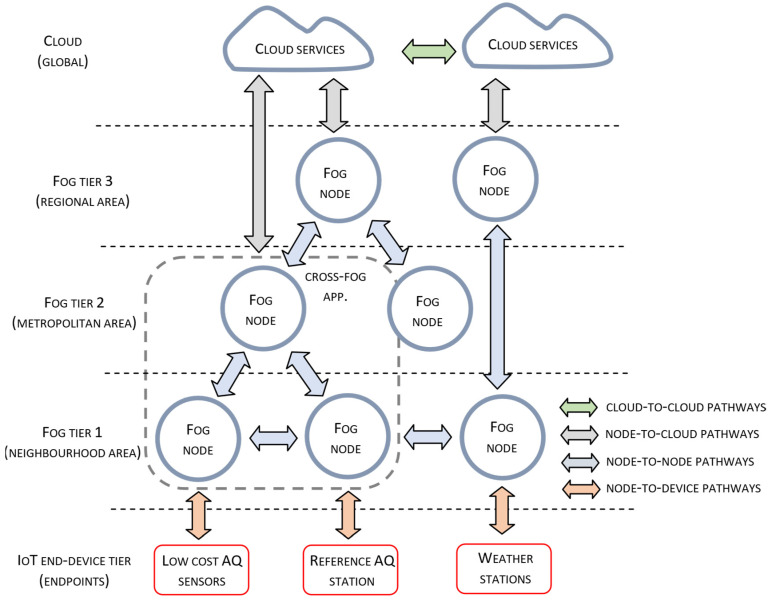
Tiered fog-based architecture for air quality monitoring.

The integration of the proposed functionalities required for building a low-cost sensing infrastructure for air quality monitoring conforms to the adopted fog computing approach. Fog computing infers that physical deployment of system elements is organized according to the tiered architectural style, as given in Figure 1. The previously defined methods for sensor side data processing, given in Section 3.1, are encapsulated as service components that reside on different tiers of system architecture. The number of fog nodes at a particular tier corresponds to the geo-locational distribution and the density of deployed measurement stations. 

Fog nodes at Tier 1 communicate with the connected sensor and/or measuring stations retrieving raw information on CO, NO, NO_2_, CO_2_, SO_2_, O_3_, PM air pollutant concentrations, as well as temperature and relative humidity measurements. After being processed by analytic services from Tier 1, measurement data and the estimated CAQI values are transferred to reference Tier 2 nodes. Analytic services at Tier 1 include a collection of sensor data processing services for sensor calibration, data correction, extractions of model parameters, etc. 

Fog nodes at Tier 2 execute several correlation-related services providing information about air quality, performing accident detection and event notification, and detection of sensor faulty operation. They also provide reference measurements data necessary for the configurations of analytic services at Tier 1 related to the sensor calibration, sensor correction, etc. Reference data can be obtained from the in-area located reference air quality monitoring station, other closely located reference stations, or provided as a result of a fusion of all available sensor measurements.

Fog nodes at Tier 3 combine information obtained from a wider district or metropolitan area. Because the nodes closer to the network core services located in the cloud have higher processing and communication capabilities, they perform more complex analytics to provide more sophisticated services related to the creation of high-resolution air pollution maps, pollution control, urban planning, land use regulation, population exposure assessment and other advanced services at the metropolitan area.

Apart from the deployment viewpoint of the AQM system, a description of system architecture, as a top-level system representation, regularly includes a description of many other structural viewpoints. We selected a software view as the one that represents the most important aspects of the system operation. In particular, our paper addresses the software view from the manageability perspective, as the cross-cutting system is responsible for ensuring successful node operation during its lifecycle. 

**Figure 2:**
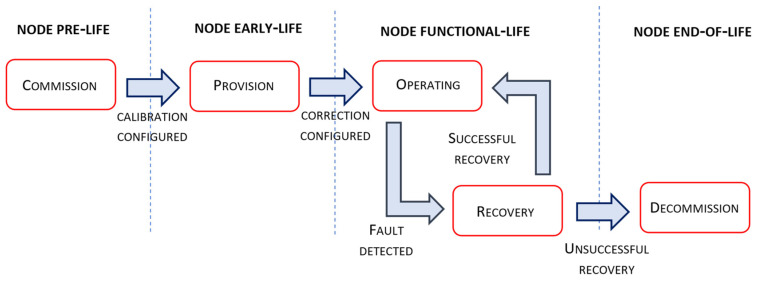
Tier 1 fog node management life cycle.

In order to bind the implementation of sensor-side processing with the service deployment and operation, we defined the manageability perspective of the edge tier. In general, fog management has a collection of responsibilities, from automated identification and discovery, advertisement of features and capabilities, provisioning of endpoint devices to more complex recovery operations. When talking about the edge tier, automation of node operation is essential during all phases of the node lifecycle because human intervention is impractical in large deployment scenarios. To enable such automation, our node management implementation complies with the finite state machine given in Figure 2. Different phases of the management life cycle are adopted from the OpenFog reference architecture [30], where transitions between lifecycle phases are aligned with the operation of introduced node services.

The Commission phase is the initial phase of a fog node lifecycle when the managed entity performs certain actions before it is ready for collecting and providing availability to its resources. These actions include the initialization of basic node functionalities related to data and sensor communication, node security and its identification, configuration and service accessibility, etc. From the manageability perspective, prior to the node provision, the sensor calibration process needs to be performed. Firstly, the node is connected to the reference area node with the node’s GPS coordinates and the information of connected sensor types. Secondly, during the configured commissioning interval, the node collects raw sensor measurements and the received reference data, extrapolated for the particular node location at the reference area node.

Based on the gathered time series of reference and raw sensor measurements, calibration model parameters are calculated according to equations 3 and 4. After the calibration service is configured with the obtained model parameter values, service operation is enabled and the node proceeds into the Provision phase. The Provision phase corresponds to the node early life, where sensor measurements are collected from the reference area node in order to train a ML model used for correction of sensor measurements influenced by particular environmental conditions. After the provisioning interval has elapsed, and the supervised ML model is trained, the correction service at the edge tier is configured and enabled, indicating node transition to its functional-life phase. 

The Operate phase of the node functional life suggests its normal operation where all identified sensor-side processing services are active, especially services used for detecting sensor faulty operation. Upon sensor fault operation detection, node operation proceeds to the Recovery phase. If the detected faulty operation corresponds to the defined alarm conditions at the edge tier, recovery operation implies notification upon the spotted node condition. In the case that the faulty operation is related to the fault indicated by the correlation service executed at the area node, automated sensor recalibration and correction model training is performed. If the implemented recovery, i.e., the recovery procedure, is not found to be effective, and the output of correlation services is still indicating sensor faulty operation, the node operation state will be transferred to the Decommission phase. The Decommission phase assumes the removal of all hardware-related node instances and interactions between software components that exist in the service execution chain. Decommission procedures are found to be more significant from the perspective of higher-tier nodes. If the recovery procedure is found to be successful, regular node operation is continued in the Operate state. 

From the architectural viewpoint, the distributed system design imposes some limitations that also need to be pointed out. From the security perspective, distributed systems are exposed to security threats because of the extensive internode communication. These threats are related to achieving data confidentiality and disabling interference and attacks by unauthorized parties. Furthermore, distributed infrastructure results in a security model with complex trust, authentication and confidentiality requirements that pervade the design of the entire system.

### 3.3. Service Operation and Deployment

**Figure 3:**
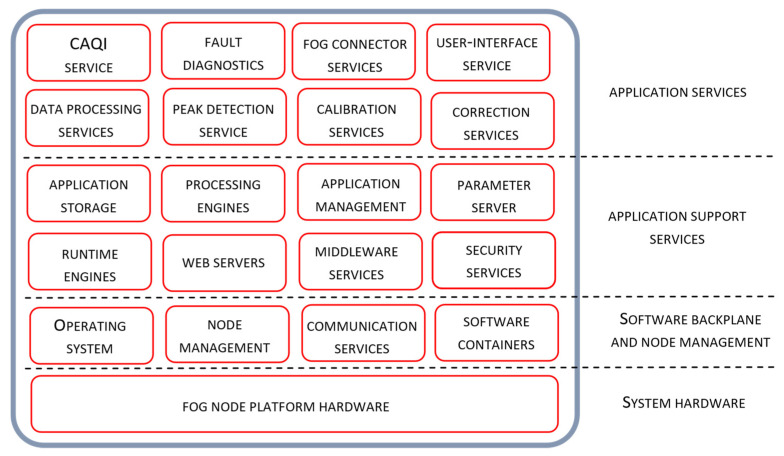
Tier 1 fog node software architecture view.

**Figure 4:**
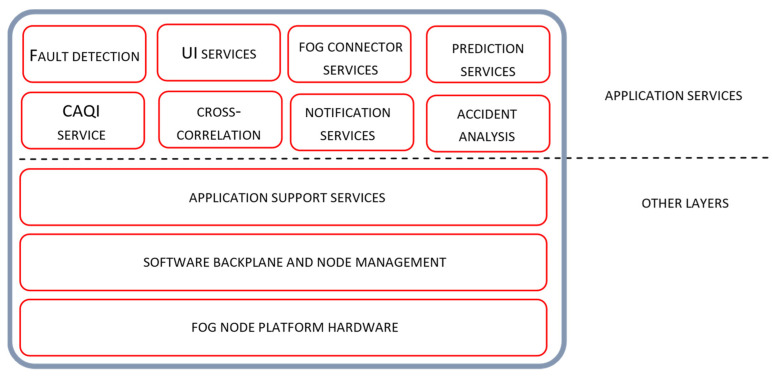
Tier 2 fog node software architecture view.

The software architecture of Tier 1 and Tier 2 nodes is presented in Figure 3 and Figure 4 respectively. Positioning of node services and the details of their individual and collaborative operation, especially from the manageability perspective, are also presented. Software architecture layers, adopted from the OpenFog reference architecture [30], include the Application Services layer, Application Support layer and Software Backplane layer with in-band node management. As given in the Figures, the tree software layers are positioned on the top of the hardware platform layer. 

The software backplane is required to run any software on the fog node and facilitate node-to-node communications, Operating system, software drivers, file system, operational and security management, etc. The Software Backplane layer orchestrates horizontal and vertical pathway communication providing data confidentiality and integrity services. The application support layer includes different software components that are commonly shared by multiple microservices. It may contain execution environments for microservices, application servers and middleware components, application data management, including different data representation formats, data encryption/decryption and persistent storage and analytic tools. The role of the application service layer is to support building a particular application. In general, fog computing applications are composed of a loosely coupled collection of microservices found at the application service layer. According to their role, these services are organized through several logical service layers including core services, support services, analytic, user interface and connector services. User interface service allows the configuration of node services, real-time access to the sensor measurements and air quality estimates, as well as access to node in-band management services. The fog connector service operates on top of the protocol abstraction layer translating the produced data into common data structures and data formats. Fog connector service is involved in internode communication over the different communication pathways across the device-to-fog-to-cloud continuum.

The majority of node services relevant for the air quality monitoring application are found at the application service layer, where particular sensor-side processing methods are encapsulated as individual node services. Related system functionality is given in the form of sequential processing steps executed through the sequence of microservices that may reside on the same or on the different architectural tiers.

The peak elimination service encapsulates the peak elimination method which is executed on Tier 1. The service inputs are the raw sensor measurements gathered from connected low-cost sensors, given in the form of time-series data. The output of the peak elimination service is given in the same form and is used as the input to the noise elimination service executing on the same node. Upon the node initialization and the configuration of service parameters, at the beginning of the commission phase of the node management life cycle from Figure 2, the service is constantly active. 

The noise elimination service encapsulates the moving average method as a method for removing noise from sensor measurements. Both the input and the output of the service are given in the same form of time series data. The service output is used as the input for the next step of the sensor-side processing sequence, where sensor calibration is performed. Upon the configuration of the noise elimination service, during the commission phase of the node life cycle, the service is periodically executed at the rate at which the input data sequence is updated. Although the parameters for this service have default values, they can be changed manually.

The sensor calibration service, located on Tier 1, encapsulates the sensor calibration method. After the service is configured with the extracted parameters of the adopted linear regression model, calibrated sensor measurements are regularly calculated according to Equation (5). The model parameters are found using Equations (3) and (4) based on the gathered time series of the observed sensor measurements and the reference sensor measurements collected from Tier 2 during the commission phase. The actual calculation is performed by the simple processing engine at the application support layer on Tier 1. Upon sensor calibration service configuration, the node continues its operation in the early-life phase, as defined in Figure 2, when the node correction service is going to be provisioned. 

As a final step in the sequence of sensor-side processing services, the sensor correction service is executed on Tier 1. This service encapsulates the RF algorithm as an ML technique used in the correction of sensor measurements. After the node carries forward its operation in the functional-life phase, the model is used to correct the sensor data samples that come out of the sensor calibration service. The configuration of correction service is performed every time the model training process on Tier 2 is finished, where model training is initiated by the transition of Tier 1 node to provision phase or its transition to the recovery phase upon the detection of sensor faults on Tier 2. All transitions between Tier 1 node life-cycle phases are defined according to the finite state machine given in Figure 2.

During the Tier 1 node operation in the functional-life phase, CAQI calculations are performed on Tier 1. The calculation is based on the gathered hourly data that come out of the correction service. CAQI service on Tier 1 calculates the pollution state for each of the air pollutants according to expression (14). The calculated CAQI index is then regularly sent to the reference area node on Tier 2, indicating very low, low, medium, high and very high pollution states. 

Besides the services related to the processing of sensor data, the application layer of the Tier 1 node contains the fault diagnostics service for detecting node operation faults related to sensor operation, data communication, power supply problems, etc. Regarding the sensor operation, the service is configured to send notification messages and alarm warnings if the missing data event, sensor communication packet loss or data out of range condition is found.

Our focus in the following discussion is to analyze the operation of relevant Tier 2 node services, given at the application service layer in Figure 4. Services are involved in the collaborative operation with the Tier 1 services, providing estimated reference sensor measurements, performing cross-correlation analysis and configuring and reconfiguring data analytic services at the edge tier. 

Regarding the operation of correction services on the Tier 1 node, related Tier 2 node functionalities include the determination of model hyperparameters and supervised model training for the selected RF algorithm used for correction services. Hyperparameters are determined through the cross-validation process performed during the Tier 1 node provision and recovery phase. Model training is performed with the sensor measurements gathered from the target edge node and the reference data extracted by Equation (7) for the particular target node location. Reference data are found as a fusion of available reference air quality stations or available sensor measurements of connected Tier 1 nodes. The trained model, with its parameters, is then forwarded to the Tier 1 correction service triggering node transition from the provision to the operate phase. Tier 2 operations regarding the setup correction services are also performed during node recovery phase or seasonally.

Detection of sensor faults related to the changes of sensor behavior is encapsulated in the form of correlation services located on Tier 2. As explained in the methodology overview section, fault detection is based on the correlation analysis of sensor measurements, gathered from target edge nodes, and reference data measurements extracted for the particular sensor location. If the fault condition, evaluated through expression (13), is fulfilled, sensor fault is detected; the corresponding edge node is transferred to the recovery state, triggering reconfiguration of calibration and correction services. It should be mentioned that the activation of fault detection services on Tier 2 are synchronized with the transition of the corresponding Tier 1 node into the operating state. The further details related to the operation of other services located on Tier 2 are out of the scope of this paper.

## 4. Experiment

This section presents a description of the experiment set-up, including node deployment and configuration, and obtained results illustrating operation and collaboration between services during the conducted experiments. The dataflow between system services during sensor-side processing is also given.

### 4.1. Experiment Set-Up

**Figure 5:**
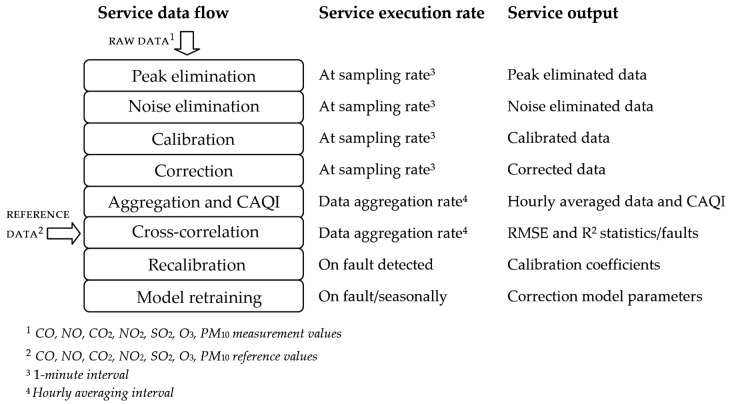
Data collection and processing flow.

Adopted methodology, system services and service collaboration are verified through the presented case study. To highlight the details of the dataflow between the system services, first we introduce the algorithm for data collection and processing, given in the form of sequential processing steps, shown in Figure 5.

Measured data from the sensors, i.e., raw values are collected from the connected low-cost measurement station, with a time resolution of 1 min, although the recommended acquisition interval can be slower. The first step in sequential processing is peak elimination, followed by a simple moving average filtering as a technique for noise elimination. After filtering and calibration, the correction of sensor data is performed. The dynamics of data processing in the preceding sequence is the same as the sampling rate of sensor data. To perform the calculation of CAQI, the mean hourly averaging of the corrected sensor measurements is performed as a part of the succeeding processing step. The same hourly averaged values and the hourly reference measurement data are used as an input of cross-correlation services. The output of this processing step is given in the form of RMSE and *R*^2^ statistics and the fault status indicating the sensor faulty operation. In case the faulty sensor state is evaluated as an output of the cross-correlation services, the recalibration of the faulty sensor is performed. Finally, seasonally or due to the detected faults, the correction model is retrained. The details of the execution of data processing services are further evaluated as a part of the presented experimental study.

**Figure 6:**
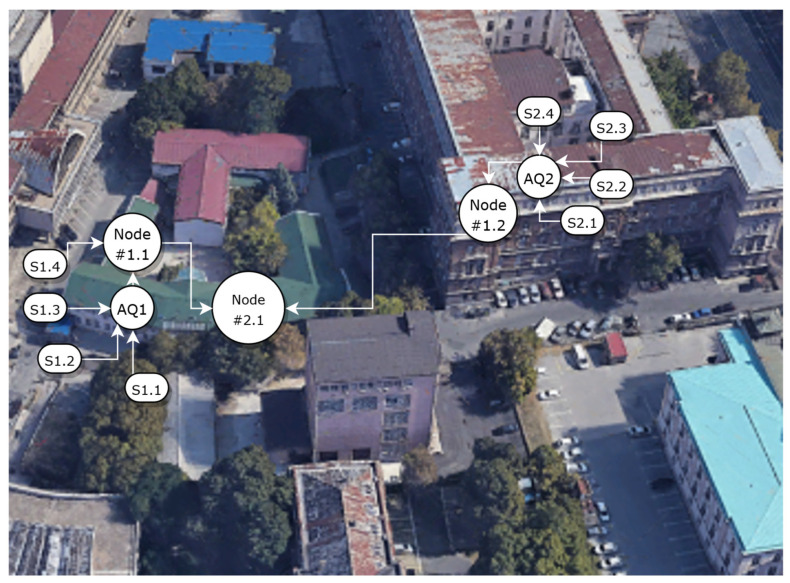
Deployment set up.

The deployment of the experimental system setup, given in Figure 6, includes a low-cost measurement station connected to Node #1.1 and nearby positioned reference measurement station Node #1.2, both connected to area Node #2.1. Although the large-scale deployment scenario is not covered within this case study, the proposed methodology and the operation of services that reside on Nodes #1.1 and #2.1, during the commissioning, early life and functional life phase of edge-tier node management life cycles were successfully verified. Further analysis of the effective system performance and the scalability of the solution requires large-scale deployment, although such properties are expected as an inherent property of the adopted distributed processing model based on fog computing.

Our case study includes several experiments conducted during the six months of system operation, from August 2020 to March 2021. As a low-cost measurement station, we used the DunavNET ekoNET device AQ10x equipped with sensors for outdoor air quality monitoring (depicted as AQ1 in Figure 6) [49]. This device contains the following air-pollution sensors for CO, CO_2_, NO, NO_2_, SO_2_, O_3_ (Alphasense, Essex, UK), PM1, PM2.5, PM10 (Plantower, District Beejing, China) and the temperature, air pressure and relative humidity sensors (Bosch BME 280, Reutlingen, Germany). Sensor measurements from the low-cost station are transferred to the fog Node #1.1 over GPRS connection, although other communication technologies, like 3G, LTE, NB-IoT, LoRa, SigFox, WiFi, and BLE, could be used. All sensor measurements are acquired at the Node #1.1 at the 1-min rate. The reference sensor contains calibrated measurements of CO, NO_2_, temperature and relative humidity (depicted as AQ2 in Figure 6.) and transfers data to the fog Node #1.2 over a GPRS connection. Nodes #1.1 and #1.2 are connected to Node #2.1 via ethernet connection and transfer data at a one-hour rate. The operation of Node #1.1 and Node #2.1 services are verified through the waveform analysis of time-series data gathered before and after being processed by a particular service or a group of services covered by three observed scenarios.

The parameter values used for configuring sensor-side processing services at Node #1.1 and Node #2.1 are listed in Table 2.

### 4.2. Experiment Results

**Figure 7:**
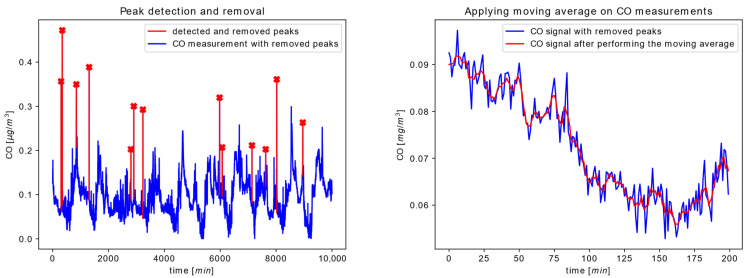
Time series data at the input and the output of the Peak elimination service (**left**) and Noise elimination service (**right**) performed on the CO sensor measurements.

In this subsection, we present the results produced by the services forming our AQM system during a real experiment. The first scenario covers the analysis of the operation of basic sensor side processing services, e.g., peak elimination and noise elimination services that reside on Node #1.1. Because the input for these services is the time series of raw sensor measurements gathered from the low-cost sensors connected to Node #1.1, the operation of services is enabled during all phases of the node life cycle, after the node-to-device communication pathway and sensor initialization. Figure 7 illustrates the peak elimination and noise elimination scenarios during the functional-life phase of node operation. Input data sequence for peak elimination service represents a time series of raw CO measurements obtained directly from the connected CO sensor, while the input sequence for noise elimination service is the output of the peak elimination service.

As one could notice from the left-side graph, although peaks occur rarely, their values are several times higher (red marked dots) than the surrounding measured values (blue line). Therefore, their influence on data-driven processing can affect the service operation and potentially lead to an erroneous conclusion. Because of this, a peak elimination service is selected as an important first step in the sequential processing of sensor data. The graph at the right-hand side of Figure 6 presents just a part of the original sequence given on the left side. It is obvious that, as expected, the moving average filtering smoothed the input data sequence (blue line) by successfully removing higher frequency information from the CO measurement data. It is also noticeable that the size of the filter length is appropriately selected because the over-smoothing and under-smoothing could not be observed from the output data sequence given by the red line. 

**Figure 8:**
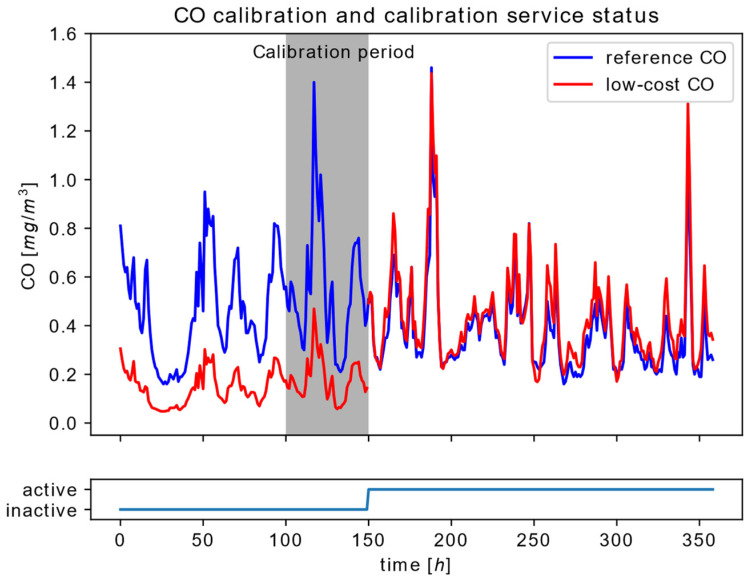
Time series data at the input and the output of the CO calibration service during the calibration period and the succeeding Node #1.1 operation in the provision phase.

The second scenario covers the analysis of Node #1.1 operation during the Commission phase and succeeding node transition to the Provision phase, as presented in Figure 8. The graph includes the co-location interval of 50 h, where the reference sensor measurements and data sequence at the output of the noise elimination service are gathered. Reference sensor measurements are obtained from reference area Node #2.1, based on the reference AQM station located in the target area. Upon the configured co-location interval has elapsed (calibration interval from Figure 8), the parameters of the linear regression model were calculated, and the calibration service was configured and enabled.

As one could notice from Figure 8, before the calibration service is enabled, there is a significant difference between the reference CO measurements and the filtered measurement obtained as an output of the noise elimination service before the calibration period. This elevates the necessity for sensor calibration because the difference between these two signals is significantly reduced after the node commission is completed and the calibration service is enabled at the time instance of 150 h.

**Figure 9:**
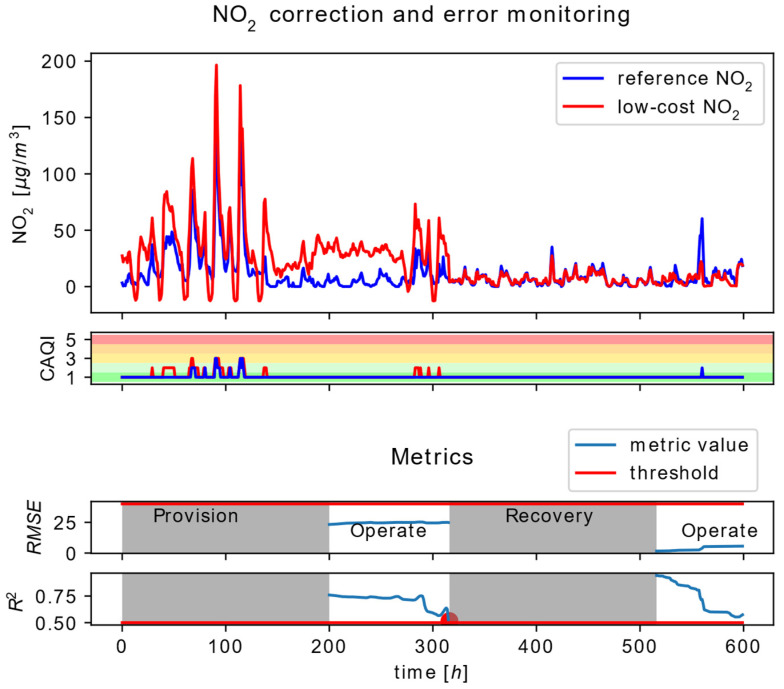
Time series data at the input and the output of the NO_2_ correction service at Node #1.1 (**top**), the output of the CAQI service at Node #1.1 (**middle**) and the statistical parameters RMSE and *R*^2^ as an output of the correlation service at the Node #2.1 (**bottom**).

Scenario three, presented in Figure 9, covers the analysis of Node #1.1 and Node #2.1 operation during Node #1.1 operation in pre-life and functional-life phase. The scenario includes the detection of Node #1.1 faulty operation and its successful recovery after the reconfiguration of the correction service. 

During the functional-life phase of Node #1.1 Operation, correction services on Node #1.1 as well as correlation services on Node #2.1 are active. Similarly, the output of the CAQI services, presented in Figure 9, is contained within the dataset transferred from Node #1.1 to Node #2.1. The correlation analysis implies monitoring of the two selected statistical parameters $R^{2}$ and RMSE, based on the estimated reference data and the gathered measurements included in the dataset received from Node #1.1. One should have in mind that the parameters are calculated based on the time sequence with n=200 samples as given in Table 1. 

As illustrated in Figure 9, at the time instance close to the 316 h, the $R^{2}$ reaches its lower limit at 0.5 (marked with a red dot), triggering the transition of Node #1.1 to its recovery phase. During the Recovery phase, based on the novel set of gathered reference and direct sensor measurements, a new ML model is trained. The recovery interval can be shortened if the reference measurements and the direct sensor measurements from the preceding training interval are buffered. A new model, with its parameters, is used for the reconfiguration of the particular Node #1.1 correction service. As given in Figure 9, after the reconfiguration of the correction service, at the beginning of the 516-h mark, recalculated $R^{2}$ and RMSE values are both in the allowed range, imposing the continued Node #1.1 operation in the operating phase.

## 5. Conclusions

The proposed fog computing approach and tiered architectural style provide an effective and flexible framework for building scalable solutions for air quality monitoring in urban areas based on low-cost sensors while at the same time enabling successful orchestration, manageability, and control of overall system resources. In addition, microservice architecture offers an adequate foundation for seamless integration of underlying methods used for sensor side data processing at the edge tier, as well as for delivering related functionalities under regular and adverse operating conditions. Collaboration of microservices during different phases of the edge-tier node life cycle are successfully tested, and its operation has been verified in the presented use case scenarios. Besides the edge-tier node commission and provision, the use case scenarios also cover the operation of higher-tier cross-correlation services for detecting erroneous sensor measurements and operational faults. The operation of such services is found not only to contribute to the improvement of overall system reliability but also in providing a basis for automated recovery services as a part of automated management capabilities, especially valuable in large scale deployment scenarios. Because the proposed architectural approach offers universal, flexible and scalable system solutions, it could be easily used for different large-scale deployment scenarios found in various smart city applications. The analysis of effective system operation performance in large-scale deployment scenarios, as well as the design of the methodology for creating high-density urban air pollution maps based on the collaboration between the group of services at the metropolitan area, is planned as a part of future work. Additionally, other available pollutant sensors’ (SO_2_, O_3_, NO, CO_2_, PM) performances with appropriate metrics (*R*^2^, RMSE) will be evaluated, alongside the analysis of the influence of wind speed and direction.

## Figures and Tables

**Table 1:** A summarized review of recent studies regarding the design, analysis and implementation of AQM methodology and architectures.

Research	Approach	Targeting	Applicability	Properties
Proposed solution	Fog based IoT	Architecture review and data processing methodology and services	Distributed fog application	Monitoring, Manageability, Scalability
[20,21,40]	Cloud-based IoT	Device design	Sensor cloud application	Monitoring
[22]	Cloud-based IoT	Device design and architecture review	Sensor cloud application	Monitoring
[23]	Cloud-based IoT	Device design and data processing algorithm	Sensor cloud application, sensor calibration	Monitoring and calibration
[24]	Cloud-based IoT	Device design and data processing algorithm	Sensor cloud application air pollution detection	Algorithm efficiency
[25]	Cloud-based IoT	Device design	Sensor cloud application	Monitoring and notifications
[26]	Cloud-based IoT	Device design and data processing algorithm	Sensor cloud application, predictive analytics	Monitoring and predictions
[35,38]	Fog based IoT	Architecture review	Sensor cloud application	Monitoring
[36]	Cloud and Edge based IoT	Device design and architecture review	Open-source sensor cloud application,	Monitoring, Scalability
[39,42]	Cloud and Edge based IoT	Architecture review	Ubiquitous sensing in smart cities	Mobile sensing, Scalability
[43]	Cloud-based IoT	Device design	Sensor cloud application	Monitoring and logging

**Table 2:** Configuration of Node #1.1 and Node #2.1 data processing services.

Service	Parameters
Peak elimination	n$=30,\;K_{1}$$=3,\;K_{2}$ = 3
Noise elimination	m = 5
Calibration	n = 50 min
Correction algorithm	n = 200 h, Random Forest algorithm
Correction algorithm parameters	100 decision trees, max features ≤ 3 features, no max depth
Cross-correlation	n$=200\;\mathrm{samples},\;t_{R^{2}}$$=0.5,\;t_{RMSE}$ = 40
CAQI calculation	t = 1 h, Number of levels = 5
